# Establishment of keratinocyte cell lines from human hair follicles

**DOI:** 10.1038/s41598-018-31829-0

**Published:** 2018-09-07

**Authors:** Tanja Wagner, Maria Gschwandtner, Agata Strajeriu, Adelheid Elbe-Bürger, Johannes Grillari, Regina Grillari-Voglauer, Georg Greiner, Bahar Golabi, Erwin Tschachler, Michael Mildner

**Affiliations:** 1 Department of Dermatology, Medical University of Vienna, Vienna, Austria; 2 Evercyte, Vienna, Austria; 3 Department of Biotechnology, University of Natural Resources and Life Sciences, Vienna, Austria; 4 Christian Doppler Laboratory for Biotechnology of Skin Aging, Vienna, Austria; 5 Department of Laboratory Medicine, Medical University of Vienna, Vienna, Austria

## Abstract

The advent of organotypic skin models advanced the understanding of complex mechanisms of keratinocyte differentiation. However, these models are limited by both availability of primary keratinocytes and donor variability. Keratinocytes derived from cultured hair follicles and interfollicular epidermis were immortalized by ectopic expression of SV40 and hTERT. The generated keratinocyte cell lines differentiated into stratified epidermis with well-defined stratum granulosum and stratum corneum in organotypic human skin models. They behaved comparable to primary keratinocytes regarding the expression of differentiation-associated proteins, cell junction components and proteins associated with cornification and formed a barrier against biotin diffusion. Mechanistically, we found that SV40 large T-antigen expression, accompanied by a strong p53 accumulation, was only detectable in the basal layer of the *in vitro* reconstructed epidermis. Inhibition of DNA-methylation resulted in expression of SV40 large T-antigen also in the suprabasal epidermal layers and led to incomplete differentiation of keratinocyte cell lines. Our study demonstrates the generation of keratinocyte cell lines which are able to fully differentiate in an organotypic skin model. Since hair follicles, as source for keratinocytes, can be obtained by minimally invasive procedures, our approach enables the generation of cell lines also from individuals not available for skin biopsies.

## Introduction

Human skin is a complex organ that provides a vital barrier to environmental pathogens and protects the body from extensive water loss^1,2^. The main barrier function of the epidermis is provided by the *stratum corneum*, which consists of denucleated terminally differentiated keratinocytes (KC)^3^. Due to their capacity to fully differentiate and to form a cornified layer, human *in vitro* skin equivalents (HSE) have been proven to be advantageous over KC monolayer cultures, especially for studying late KC differentiation and epidermal barrier function^4–6^. These models consist of primary human KCs that are seeded on a dermal matrix^6,7^ which then are brought to the air-liquid interface and form an epidermis that closely resembles that of human skin^8–10^. Although such models are widely used in dermatological research they have several drawbacks, such as high donor-variability, short *in vitro* lifespan of KC and limited availability of donor tissue^11–13^. These restrictions could potentially be overcome by the use of immortalized KC cell lines which bypass short *in vitro* lifespan and allow the generation of multiple uniform HSE from one donor cell line^14,15^. To obtain such cell lines cellular DNA damage response and telomere shortening have to be avoided^16–18^. In the past different approaches to KC immortalization, e.g. overexpression of telomerase and inactivation of cell cycle regulatory genes have been used^19–28^. However, most of the available immortalized KC cell lines, including spontaneously immortalized tumor lines, either lost their capacity to differentiate, resulting in poor epidermal morphology and barrier function, or still have limited population doublings^23,25,26,29–31^.

The main sources for primary KC are skin biopsies derived from plastic surgery or neonatal foreskin. Besides the interfollicular epidermis also the outer root sheath of hair follicles provides easily accessible KC that can be harvested under minimally invasive conditions^32–34^. As has been shown previously, KCs derived from hair follicles can be readily used to establish HSE^32,34,35^. However, the use of primary hair follicle-derived KC for HSE is again restricted by limited cell doublings^35^.

Here we demonstrate that ectopic expression of the SV40 together with human telomerase reverse transcriptase (hTERT) generates immortalized KC cell lines from hair follicle KC that can be used to study KC differentiation in fully differentiated HSE.

## Results

### Establishment of human KC cell lines derived from hair follicles and interfollicular epidermis

**Figure 1:**
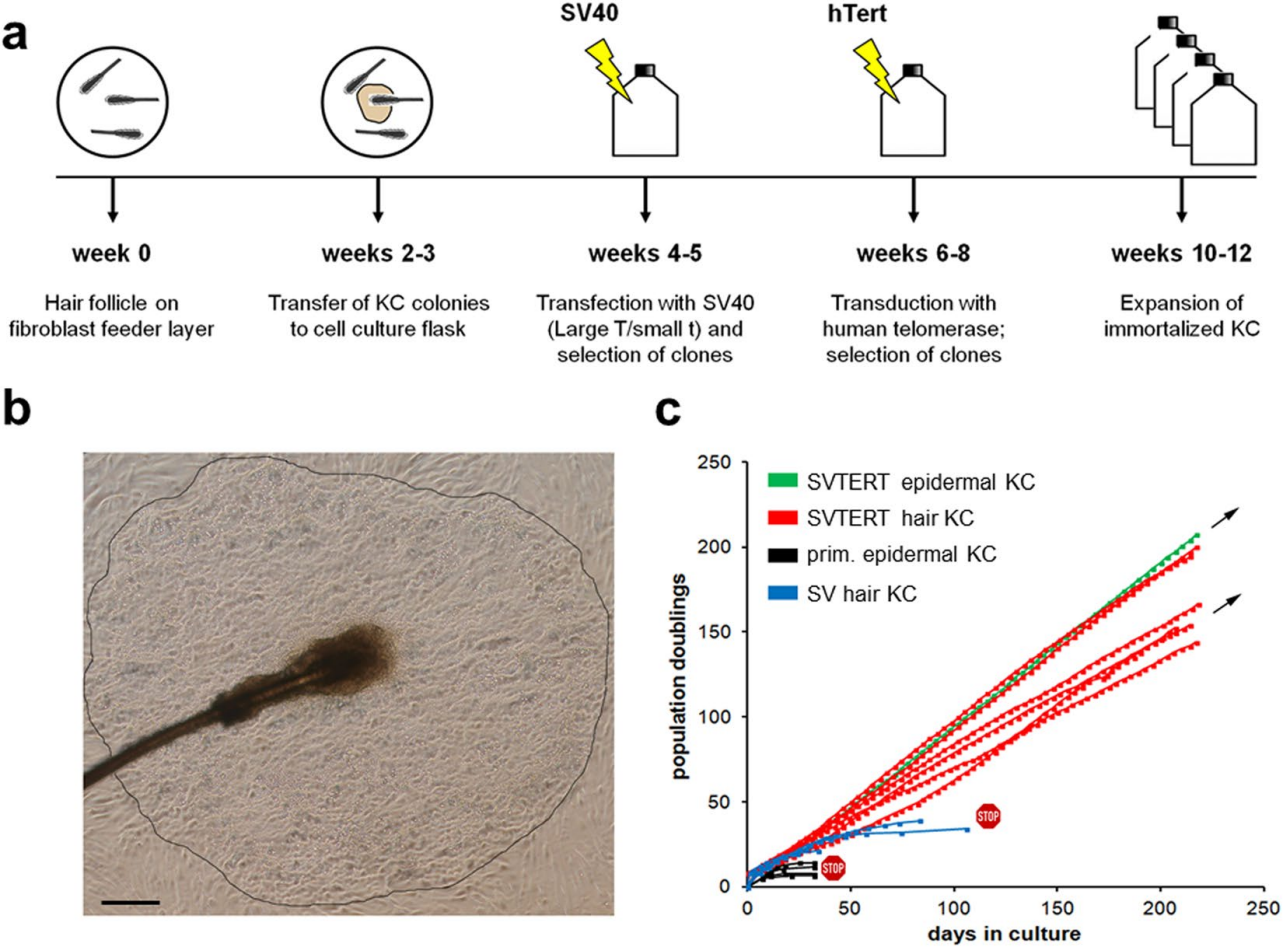
Establishment and growth characteristics of human KC cell lines. For KC isolation plucked hair roots were placed on growth inactivated fibroblasts (**a**), round KC colonies that formed after 2–3 weeks (**b**) were transferred to cell culture flasks and the cells were subjected to different immortalization protocols (**a**). Growth curves show the extension of KC life span after SV40 transfection (SV; blue lines) and the establishment of stable KC cell lines after SV40 transfection and subsequent hTERT transduction (SVTERT; green and red lines) (**c**). Scale bar = 480 µm.

KC from human hair follicles were generated as depicted in Fig. 1a. Individual scalp hairs were plucked with a forceps and placed in tissue culture on a feeder layer of growth-arrested fibroblasts. After 2–3 weeks, dense KC colonies formed around the hair roots (Fig. 1b) which were removed from the feeder layer by selective trypsinization. KC from abdominal epidermis were isolated according to a standard protocol (see Materials and Methods). After sub-culturing into cell culture flasks, KC of both sources were immortalized by transfection of a cDNA encoding SV40 and were subsequently transduced with an expression vector for hTERT (Fig. 1a). Repeatedly about 10% of the KC survived the immortalization protocol and kept growing continuously. As compared to primary cells with a life span of 30 days and 12 doublings, transfection of KC with the SV40 alone tripled their life span to up to 100 days and 40 doublings before they entered senescence. Transfection with SV40 and subsequent transduction with hTERT resulted in KC cell lines (SVTERT KC) with a stable growth rate for more than 200 days (Fig. 1c). The morphology of monolayer cultures was similar for primary KC and SVTERT KC derived from epidermis and hair follicles (Figure S1). In total one cell line from interfollicular epidermis and 6 hair-derived cell lines were established and all cell lines behaved comparably.

### SVTERT KC show typical KC differentiation in monolayer cultures and in an *in vitro* reconstructed epidermis

**Figure 2:**
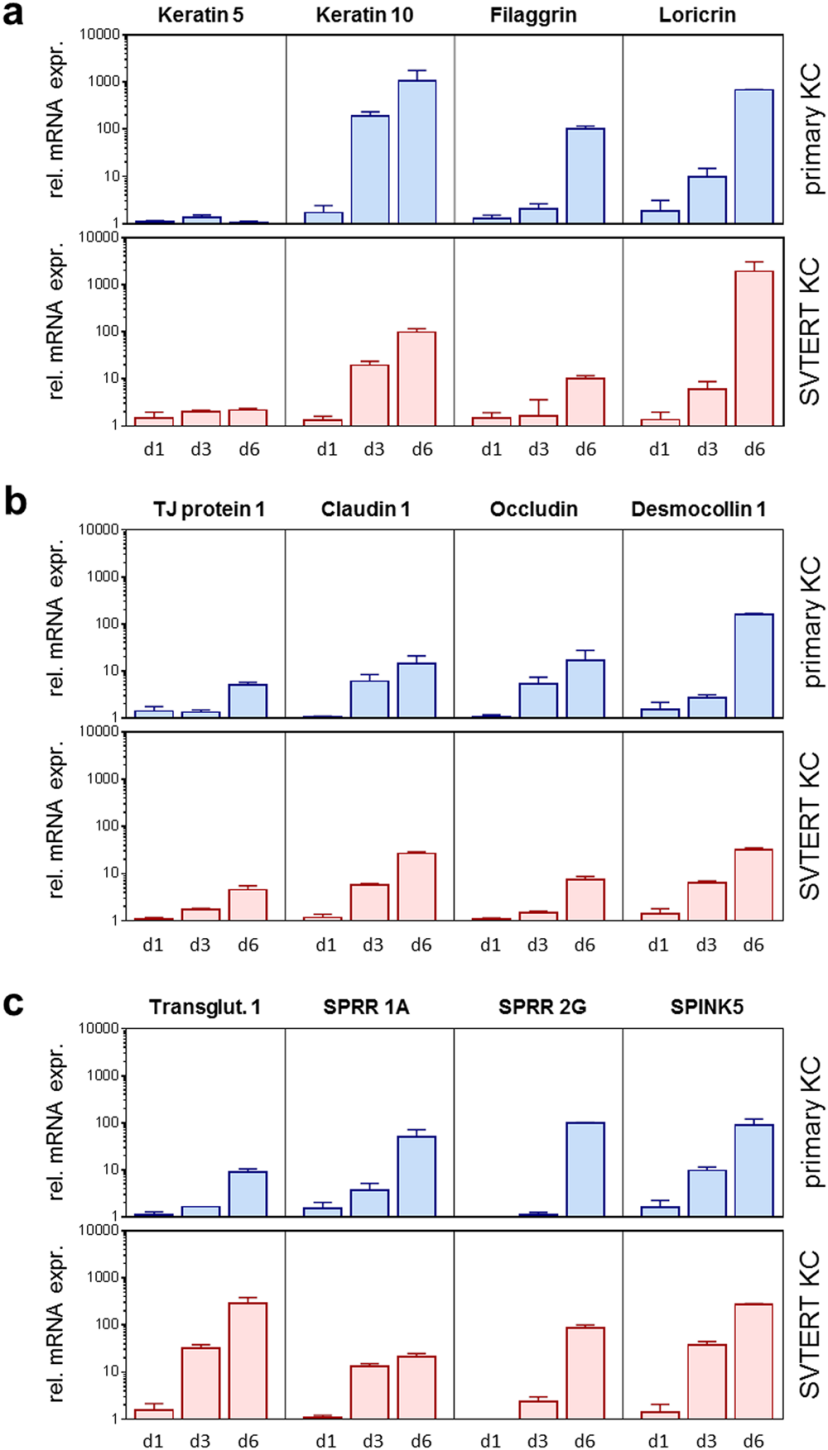
Primary and SVTERT KC differentiate in monolayer culture. Primary and SVTERT KC derived from epidermis were cultured post-confluent for six days and mRNA expression levels were analyzed by real-time PCR at the indicated time points. Primary and SVTERT epidermal KC showed no induction of the basal marker keratin 5 and showed upregulation of differentiation-associated proteins keratin 10, filaggrin and loricrin (**a**). Similarly, the cell junction components tight junction protein 1, claudin 1, occludin and desmocollin 1 (**b**) and the cornification and desquamation associated fators transglutaminase 1, small proline rich protein 1 A and 2 G and serine protease inhibitor Kazal-type 5 SPINK5 (**c**) were induced during differentiation in primary and SVTERT KC.

**Figure 3:**
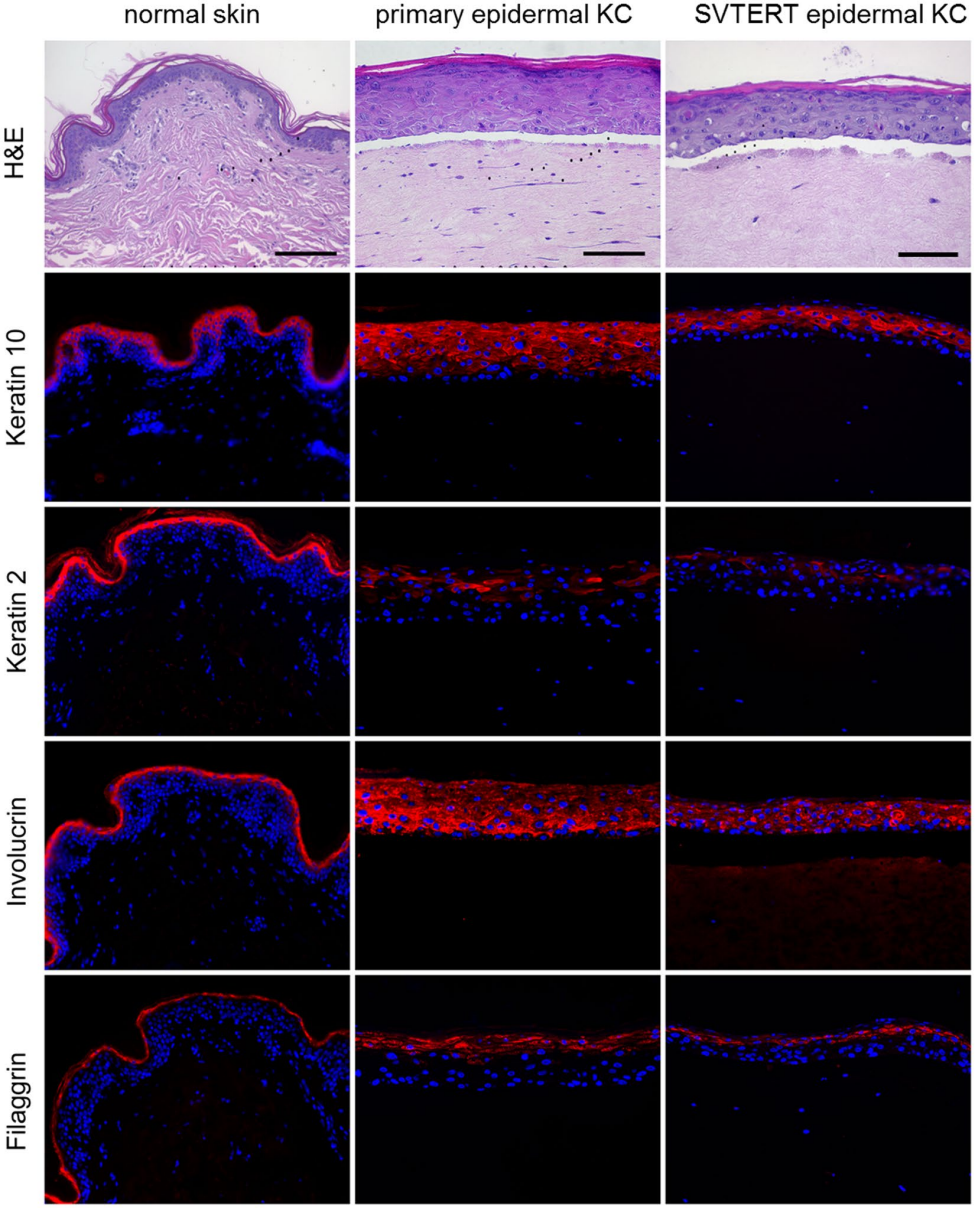
Primary and SVTERT epidermal KC form fully differentiated skin equivalents. Normal abdominal skin and human skin equivalents (HSE) at day seven were analyzed by hematoxylin and eosin (H&E) and immunfluorescence staining. HSE with primary and SVTERT KC developed a multilayered epidermis with intact stratum granulosum and stratum corneum resembling human skin. The differentiation associated proteins keratin 10, keratin 2, involcurin and filaggrin were detected by immunofluorescence staining, both in normal skin and HSE cultures. One representative experiment out of three is shown; Scale bar = 120 µm.

**Figure 4:**
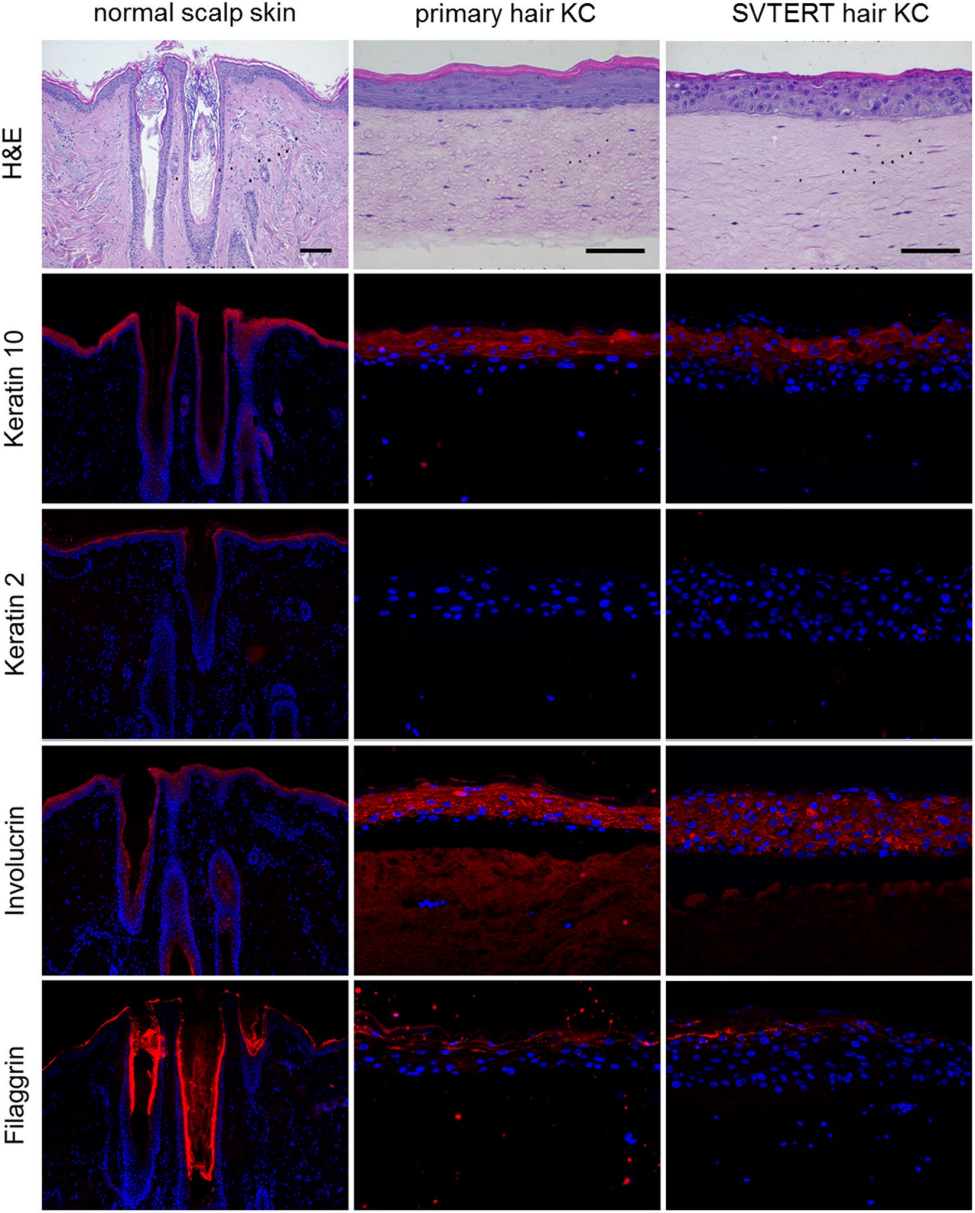
Primary and SVTERT hair KC form completely differentiated skin equivalents. Normal scalp skin and HSE at day seven were analyzed by hematoxylin and eosin (H&E) and immunfluorescence staining. HSE developed a multilayered epidermis with intact stratum granulosum and stratum corneum resembling human skin. The differentiation associated proteins keratin 10, involcurin and filaggrin were detected by immunofluorescence staining, both in scalp skin and HSE cultures. keratin 2 was detected in scalp skin, but not in SE cultures. One representative experiment out of three is shown; Scale bar = 120 µm, for scalp 240 µm.

**Figure 5:**
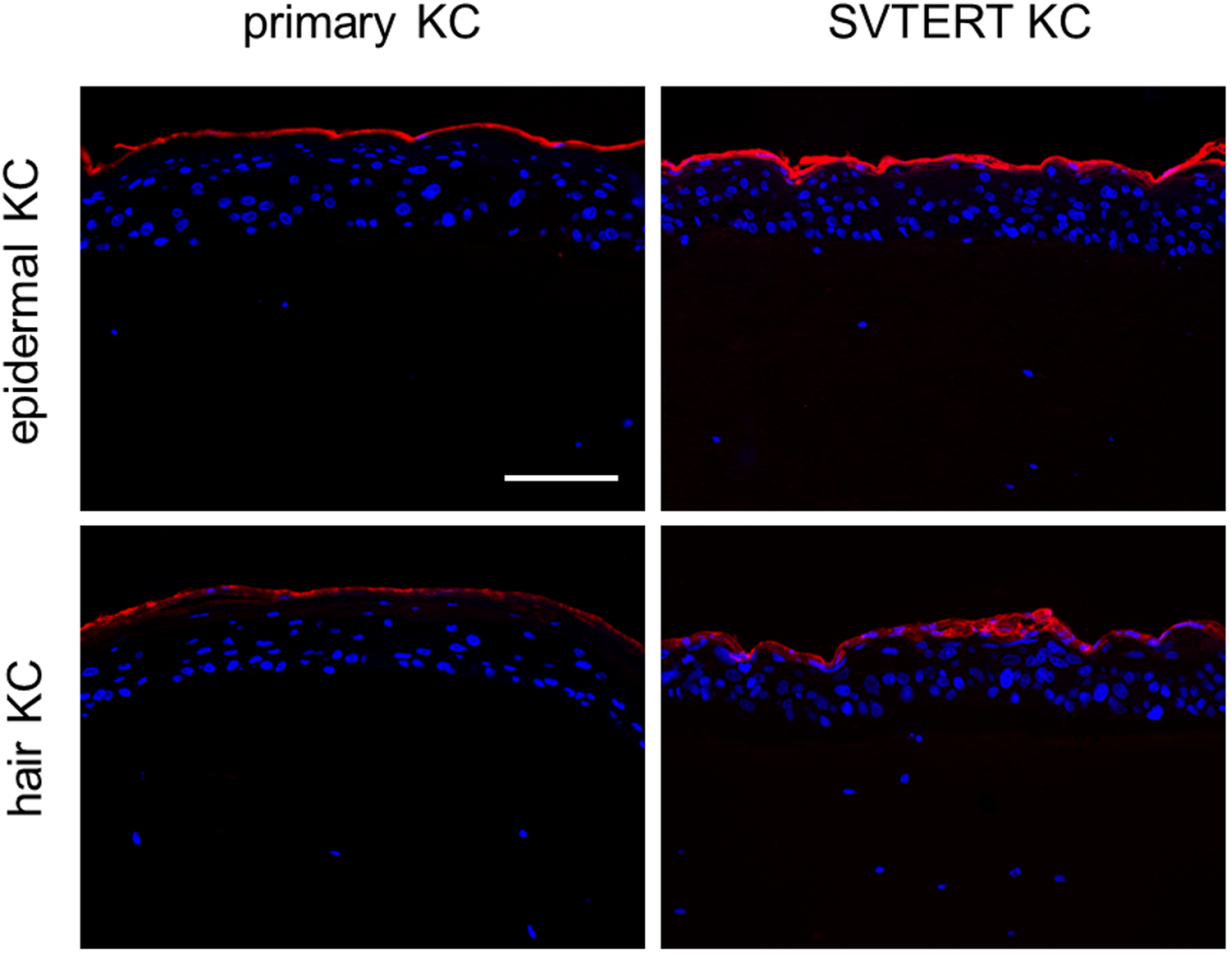
Biotin does not diffuse into the stratum corneum of skin equivalents with primary and SVTERT KC. Biotin was added to the surface of fully developed human skin equivalents (HSE). In all HSE biotin could not penetrate the stratum corneum into the deeper layers within 60 min. Scale bar = 120 µm.

Primary KC and SVTERT KC were differentiated in monolayer cultures by growing them at post-confluency for up to ten days. The morphology of the primary KC and SVTERT KC changed similarly during the differentiation process (data not shown). qPCR analysis revealed comparable regulation of differentiation-associated proteins (keratin 5 and 10, filaggrin and loricrin; Fig. 2a), cell junction components (tight junction protein 1, claudin 1, occludin and desmocollin 1; Fig. 2b) and factors associated with desquamation and cornification (transglutaminase 1, small proline rich proteins 1 A and 2 G and serine protease inhibitor Kazal-type 5 SPINK5; Fig. 2c) during the differentiation process of primary KC and SVTERT KC. Exemplarily, the regulation of Keratin 10 (KRT10) was confirmed also on the protein level (Figure S2). To further study the differentiation potential of SVTERT KC, *in vitro* HSE were established and analyzed by conventional histology and by immunostaining. The morphology of HSE prepared with epidermal- (Fig. 3) and hair-derived primary and SVTERT KC (Fig. 4) was comparable and resembled normal human epidermis with regard to the formation of the different epidermal layers and the expression of epidermal differentiation markers (Figs 3 and 4, Fig. S3). In HSE from epidermis-derived KC and SVTERT KC KRT10 and filaggrin expression patterns were similar to that of normal human skin (Fig. 3). However, KRT2 and involucrin, which showed continuous staining pattern in the stratum granulosum of normal epidermis were expressed in HSE focally and in all epidermal layers respectively (Fig. 3). HSE prepared from hair-derived KC and SVTERT hair KC expressed KRT10, involucrin and filaggrin similarly to the above, but consistently lacked KRT2 expression (Fig. 4). In addition, biotin diffusion into the layers beneath the stratum corneum was comparably absent in primary and SVTERT KC (Fig. 5). After being kept in culture for more than 200 doublings both epidermis- and hair-derived SVTERT KC lost the ability to form well-differentiated HSE. Rather, these HSE lacked a stratum granulosum, did no longer form a distinct stratum corneum and showed very low expression and abnormal distribution of KRT10 and involucrin (Figure S4). The expression patterns of Ki67 in some basal keratinocytes in HSE and β-galactosidase as marker for senescence were not modulated in SVTERT KC cultured for more than 200 doublings (Figure S5). Chromosomal changes in >200 doublings SVTERT KC (5% diploid, 75% hypodiploid, 20% hypotetraploid) were minimal compared to <100 doublings SVTERT KC (8% diploid, 81% hypodiploid, 12% hypotetraploid).

### SV40 large T-antigen and p53 expression is lost in the suprabasal cell layers of *in vitro* reconstructed epidermis

**Figure 6:**
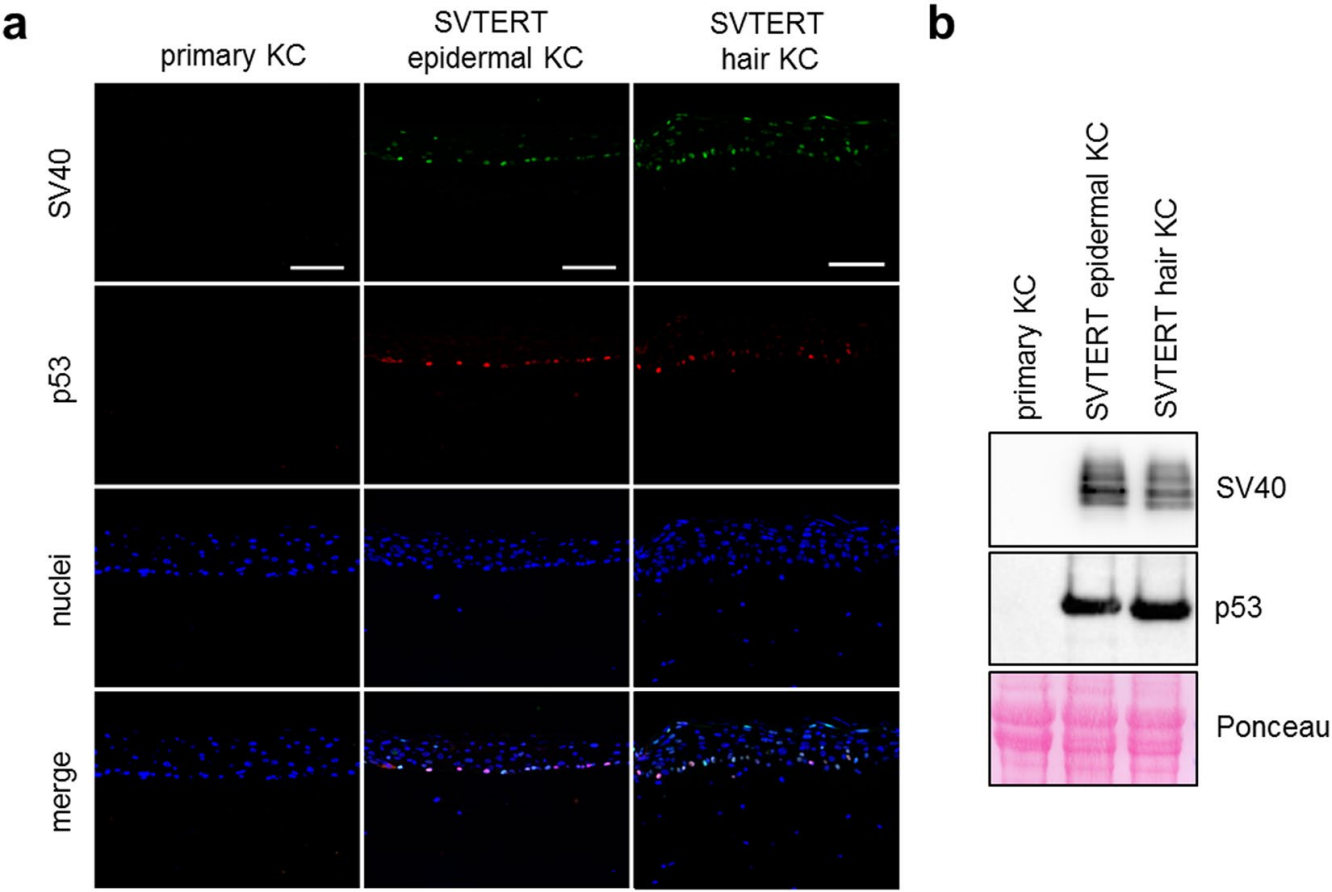
Skin equivalent cultures with SVTERT KC show p53 expression limited to the basal layer. HSE cultures from primary and SVTERT skin and hair KC were established and analyzed at day seven by immunfluorescence staining (**a**) and Western Blot (**b**). In primary KC no p53 and SV40 large T-antigen expression was detected. In SVTERT KC the expression of SV40 large T-antigen was restricted to the basal and granular cell layer; p53 was only detected in the basal cell layer. One representative experiment out of three is shown and Ponceau S stainings was used as loading control in Western Blots; scale bar = 120 µm.

The immortalization protocol included the introduction of the SV40 large T-antigen resulting in the accumulation and inactivation of p53. Therefore, normal differentiation of SVTERT KC in HSE was quite unexpected. Immunofluorescence (Fig. 6a) and Western blot analysis (Fig. 6b) confirmed that in HSE established with primary KC SV40 large T-antigen and p53 expression was not detected. Interestingly, in HSE prepared with epidermis-derived SVTERT KC SV40 large T-antigen staining accompanied with accumulation of p53 was detected in cell nuclei mainly in the basal epidermal cell layer but not in most of the suprabasal cells (Fig. 6a). In hair-derived SVTERT HSE SV40 large T-antigen expression was also detectable in some cells of the last living layer but was not accompanied by p53 accumulation (Fig. 6a). Both p53 and SV40 large T-antigen showed a strong signal in Western blot analysis (Fig. 6b) and a phospohokinase proteome profiler (Figure S6), confirming the immunostaining results. SVTERT KC kept in culture for more than 200 doublings, did no longer downregulate SV40 large T-antigen expression in suprabasal layers and p53 was also detectable in KC of several layers (Figure S4).

### DNA methylation is involved in the down-regulation of SV40 large T-antigen and p53 expression in skin models derived from SVTERT KC cell lines

**Figure 7:**
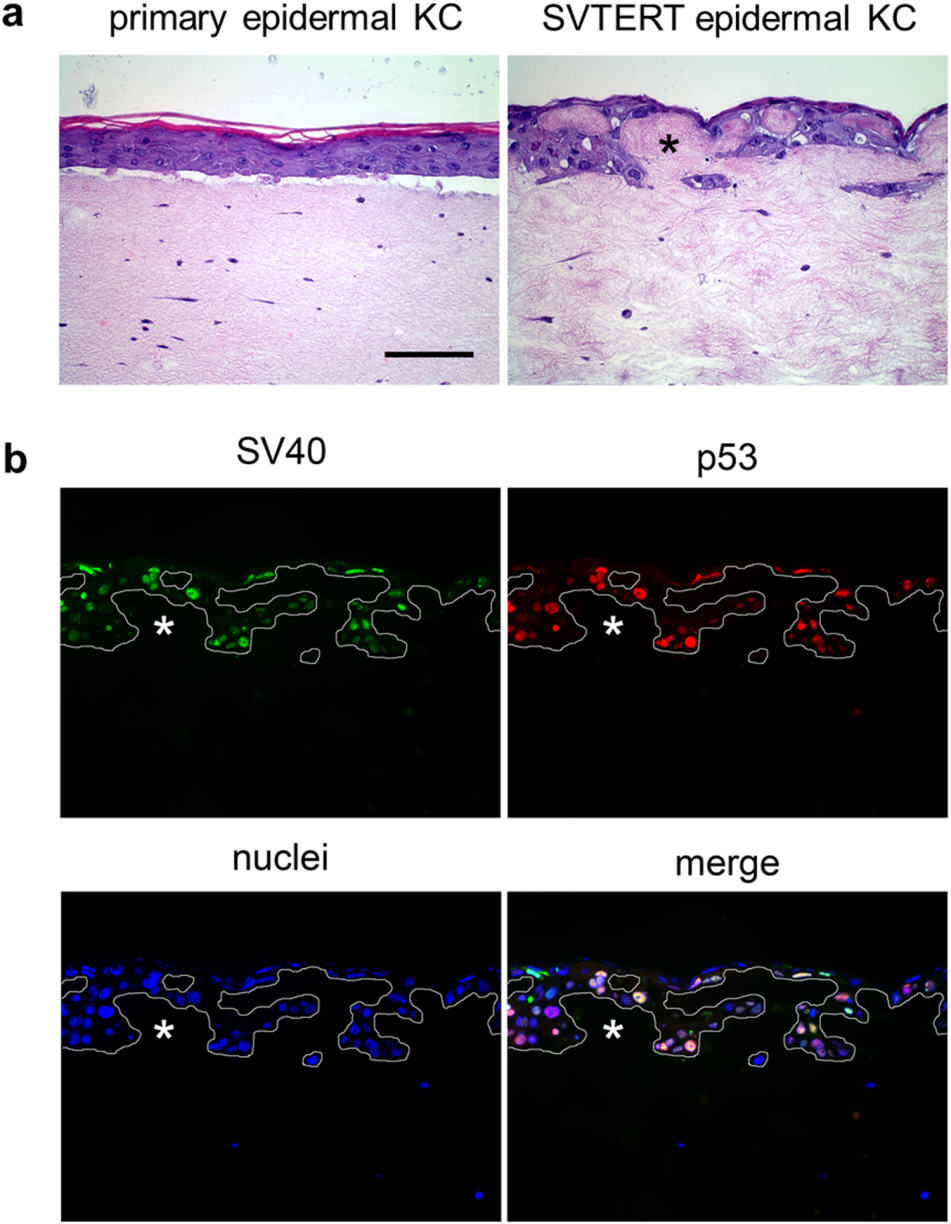
Inhibition of DNA methylation results in loss of normal epidermal architecture in skin equivalents with SVTERT KC. HSE cultures with primary and SVTERT skin KC were incubated with 10 µM of DNA methylation inhibitor 5-Aza-2′-Deoxycytidine and analyzed at day seven. Hematoxylin and eosin (H&E) staining showed no effect on primary KC, but completely disturbed epidermal morphology for SVTERT KC (**a**). Immunfluorescence staining showed that SV40 large T-antigen and p53 was not confined to the basal layer, but was detected in all layers (**b**). Bar = 120 µm.

To unravel the mechanisms for SV40 large T-antigen down-regulation in the suprabasal cell layers of SVTERT KC derived HSE, we cultured HSE in the absence and presence of MG132 and bafilomycin, inhibitors for the proteasomal and autophagy-lysosomal degradation pathway, respectively. Inhibition of proteasomal degradation in HSE neither affected epidermal KC differentiation (Figure S7a) nor SV40 large T-antigen expression and p53 accumulation (Figure S7b). In contrast, inhibition of the autophagy-lysosomal pathway by culturing HSE in the presence of bafilomycin, strongly affected KC differentiation and the formation of the cornified layer (Figure S8a). However, the loss of SV40 large T-antigen expression and p53 accumulation in the suprabasal layers was not affected (Figure S8b). We next investigated whether inhibition of DNA methylation by the specific DNA methylation inhibitor 5-Aza-2′-Deoxycytidine affects SV40 inhibition in the suprabasal epidermal cell layers. Cultivation of HSE in the presence of the DNA methylation inhibitor did not affect KC differentiation and epidermal development when using primary epidermal KC (Figure 7a, left panel). However, when the same treatment was applied to HSE established with SVTERT epidermal KC we found an entirely disorganized accumulation of KC, a lack of stratification and invasion into the collagen matrix (Fig. 7a, right panel). Both SV40 large T-antigen and p53 were prominently expressed in more than 90% of the KC (Fig. 7b).

## Discussion

Normal KC-differentiation is a complex program which involves tight regulation of gene expression as well as post-transcriptional events^2,36^. To study KC-differentiation *in vitro*, skin equivalent models have been developed which reflect most of the biological processes occurring in human epidermis *in vivo*^5–10^. However, these models suffer from two major limitations: i) to obtain primary KC, skin biopsies are needed which is a considerable burden for patients, and ii) primary KC have a limited life span, of approximately 15 population doublings, allowing only a limited number of experiments to be performed with one donor. In the present study we tried to overcome both problems by establishing KC cell lines from primary hair follicle-derived KC cultures and compared them to cell lines established from primary epidermal KC.

In analogy to a previous publication by Wang and colleagues^37^ we were able to grow pure KC cultures from plucked hair follicles within 2–3 weeks to quantities sufficient for the establishment of a few HSE. Initially, we compared these primary hair follicle-derived KC to the widely used epidermis-derived KC. When cultivated in monolayer, hair follicle-derived KC showed a similar morphology as well as growth- and differentiation behavior as compared to KC prepared from healthy epidermis. Histological analysis of HSE prepared with hair-derived KC showed a multi-layered stratified epidermis, also comparable to that of HSE established with epidermal KC. Our results are in line with previously published data^32,34,35^, confirming the potential of hair-derived KC to fully differentiate *in vitro* similar to epidermal KC. Our models also displayed typical expression and distribution of most differentiation-associated proteins, such as KRT10, involucrin and filaggrin with the notable exception of KRT2. This type II keratin is present in the granular layer of the epidermis and was readily detectable in epidermal KC HSE but was not detected in the hair follicle-derived skin models, confirming that this keratin is not expressed in human outer root sheath KC^38,39^. We have previously demonstrated that KRT2 is a binding partner for KRT10 in interfollicular epidermis of the mouse, and lack of KRT2 led to irregular cytoskeleton formation and KRT10 aggregation^39^. Such KRT10 aggregates were not detectable in our hair-KC-derived skin models. Furthermore, KRT2 mutations in humans cause ichthyosis bullosa of Siemens^40–42^, a disease characterized by cell lysis occurring in the spinous and granular layers of the epidermis^43^. Thus, our data suggest that in outer root sheath KC KRT2 is substituted by other keratins, allowing normal cytoskeleton formation and KC differentiation. This finding suggests that it might be worthwhile to consider treating ichthyosis Siemens patients by transplanting hair-derived KC on the affected areas in an autologous setting.

A single step immortalization approach based on SV40 transfection generated cell lines that fully differentiated in a HSE model (not shown). However, the *in vitro* lifespan was limited to 40 population doublings. In contrast, the two-step immortalization protocol combining SV40 transfection with hTERT transduction resulted in immortalization of KC with a lifespan exceeding the single immortalization protocol by far. Both epidermal and hair-derived KC showed linear growth rates over the investigation period of 200 culture days. This is in line with a previous report describing the immortalization of KC of the oral mucosa^27^. In addition, skin models established with cell lines from epidermal and hair-derived KC showed expression patterns of the differentiation specific markers KRT2, KRT10, involucrin and filaggrin comparable to those of their respective primary KC. Although, in contrast to normal skin, barrier formation in HSE models is fundamentally limited, we observed an intact biotin diffusion barrier in HSE from both epidermis and hair derived KC. These findings were somehow unexpected, since other widely used KC cell lines, such as HaCat and A431 cells, have defects in the KC differentiation program, leading to imperfect terminal KC differentiation in an organotypic skin model^29^ (Figure S9A). One possible reason for the incomplete differentiation process of HaCaT cells and other KC cell lines might be that they carry functional mutations in the p53 gene^14,27,44^. To test whether inactivation of p53 would also have consequences in our model, we performed experiments in the presence of a chemical p53 inhibitor, showing that p53 inhibition indeed severely disturbed the formation of the cornified layer in skin models (Figure S9B). Skin models with SVTERT cells (<100 PDs) expressed SV40 large T-antigen only in the basal layer of the reconstructed epidermis and p53 accumulation was observed exclusively in the basal layer of the HSE. These data imply that the down-regulation of SV40 large T-antigen expression in the suprabasal layers of the epidermis is necessary for normal p53 function, and KC differentiation. The fact that SV40 large T-antigen expression is down-regulated in suprabasal SVTERT KC is striking, and similar observations have not been reported before. The possibility that proteasomal and lysosomal degradation were involved in the loss of SV40 large T-antigen expression^45–47^ was excluded by our finding that neither inhibition of the proteasomal-, nor blocking of the lysosomal/autophagic degradation pathways prevented SV40 large T-antigen down-regulation. By contrast, inhibition of DNA-methylation by 5-Aza-2′-Deoxycytidine resulted in SV40 large T-antigen expression throughout all epidermal layers, strongly suggesting that methylation of SV40-DNA in the suprabasal KC layers inhibited SV40 large T-antigen expression. It is well known that methylation of viral sequences integrated in host genomes is a strong cellular defence mechanism^48^. Our finding of a methylation dependent silencing of SV40 large T-antigen expression in the suprabasal layers of KC leads us to speculate that a differentiation dependent silencing of viral genes and promoters in the epidermis might represent a first-line defence against viral infections of KC, or may help to limit the spread of viruses within the epidermis. Our cell lines and HSE might represent valuable model systems to study such a defence system in the human skin.

When SVTERT KC were cultured for more than 200 population doublings, they showed incomplete KC differentiation which was accompanied by a lack of SV40 inhibition. Since DNA-methylation is known to decrease in mammalian cells during ageing *in vivo*^49^, a reduction of DNA-methylation might also occur in aged cells in culture. In addition, it has been reported that stability of other cells overexpressing telomerase is also limited to about 100–120 population doublings^50,51^. However, further experiments are needed to fully elucidate the interaction of DNA-methylation and KC-differentiation. Intriguingly, we only found minimal changes in the karyotype of low and high passage SVTERT KC, showing some minor chromosomal alterations already in the low passage SVTERT cell lines (8% diploid, 81% hypodiploid, 12% hypotetraploid). Nevertheless, chromosomal changes were much less pronounced than in low passage HaCaT cells (0% diploid, 58% hypodiploid, 42% hypotetraploid)^52^, suggesting a high genomic stability of the SVTERT cell lines.

In summary, we present a two-step immortalization protocol using SV40 and hTERT resulting in KC cell lines exhibiting growth and differentiation characteristics similar to primary cells, especially with regard to the formation of fully-differentiated skin equivalent models. Indeed, one of the skin cell lines (NHEKSVTERT5_3) has already been used as a stable model system for long-term exposure to toxic substances such as arsenic^53^. Importantly, Weinmüllner and co-workers could demonstrate in this study that the generated SVTERT cell lines are not tumorigenic and only form transient tumors when injected into SCID mice.

Moreover, we showed that hair follicles are an excellent alternative source for primary KC and KC cell lines and behave similar to epidermal KC. This is of practical relevance, since hair follicles can be obtained by minimally invasive procedures and therefore allows the generation of cell lines from individuals not available for (repeated) skin biopsies. Hair follicle-derived KC cell lines of patients with skin diseases might therefore represent a useful tool to study pathomechanisms of skin diseases.

## Methods

### Ethics statement

Human material was obtained in compliance with local laws and regulations and the responsible ethical committee (Medical University of Vienna) has approved all experimental procedures. Primary cells were only isolated from human material with signed informed consent from the donor. The privacy and autonomy of the donors has been protected.

### Antibodies

All antibodies used are listed in Tables 1 and 2.

**Table 1:** List of used primary antibodies.

Antigen/Clone	Western blot	Immunofluorescene staining	Host species	Company
Filaggrin/15C10)	—	1:200	mouse	Neuromics^i^
Involucrin/SY5	—	1:2000	mouse	NeoMarkers^ii^
Keratin2/5091	—	1:200	mouse	Acris^iii^
Keratin10/PRB-159P)	1:1000	1:1000	rabbit	Covance^iv^
Ki67 (ab15580)		1:1000	rabbit	Abcam^v^
p53 (ab31333)	—	1:2000	rabbit	Abcam^v^
p53 (Clone DO-1)	1:500	—	mouse	Merck EMD Millipore^vi^
SV40 large T-antigen (PAb416)	1:100	1:200	mouse	Abcam^v^

Company address: ^i^Edina, MN, USA; ^ii^Fremont, CA, USA; ^iii^Herford, Germany; ^iv^Princeton, NJ, USA; ^v^Cambridge; UK, ^vi^Deramstadt, Germany.

**Table 2:** List of used secondary antibodies.

Secondary antibody	Western blot	Immunofluorescence staining	Company
Alexa fluor® 546 goat anti- rabbit IgG (H + L)	—	1:500	Invitrogen^i^
Alexa fluor® 546 goat anti- mouse IgG (H + L)	—	1:500	Invitrogen^i^
Alexa fluor® 488 goat anti- mouse IgG (H + L)	—	1:500	Invitrogen^i^
ECL™ Anti-mouse IgG, Horseradish Peroxidase linked whole antibody (from sheep)	1:10000	—	GE Healthcare^ii^
Blotting Grade Goat Anti-Rabbit IgG (H + L), Horseradish Peroxidase conjugate	1:10000	—	BioRad^iii^

Company address: ^i^Carlsbad, CA, USA; ^ii^Little Chalfont, United Kingdom; ^iii^Hercules, CA, USA.

### Cell isolation and culture

Epidermal keratinocytes were isolated from human abdominal skin as follows. Briefly, epidermis was separated from dermis after overnight dispase II (2.4 U/ml; Roche, Mannheim, GER) treatment and KC single cell suspensions were prepared by trypsin (Lonza, Walkersville, MD, USA) digestion at 37 °C for 10 minutes.

KC from hair follicles were isolated according to a published protocol (34). Approximately 3–5 plucked hairs with roots were placed in a 6-well on a feeder layer of fibroblasts growth-inactivated with Mitomycin C (2 µg/ml; Sigma-Aldrich, St. Louis, MO, USA) in a growth medium containing 65% DMEM (GE Healthcare, Little Chalfont, UK), 25% keratinocyte growth medium-2 (KGM-2, Lonza, Basel, CH) and 10% fetal calf serum supplemented with Triiodo-L-thyronine (Sigma-Aldrich) and Penicillin/Streptomycin (Gibco, Carlsbad, CA, USA). KC colonies were selectively trypsinized from the feeder layer and subcultured in KGM-2 for further experiments and cell immortalization.

### Immortalization of keratinocytes

KCs were immortalized at Evercyte (Vienna, Austria). KCs derived from human skin and from human hair follicles were transfected using Lipofectamine 2000 (Life Technologies, Carlsbad, USA). 25 μl Lipofectamine 2000 was mixed with 500 μl Opti-MEM I reduced serum medium (Thermo Fischer Scientific). After incubation at room temperature for 5 min, a mixture of 10 μg plasmid DNA pDEPT (containing SV40 large T and small T) and 500 μl Opti-MEM I reduced serum medium was added to the Lipofectamine mixture and incubated for additional 20 min at room temperature. The solution was then added to 5 ml Opti-MEM I reduced serum medium and transferred to the KCs. After incubation at 37 °C for 4 h, the transfection solution was removed and fresh KGM-2 medium was added to the KCs. After 2 to 3 weeks after transfection KCs were transduced with hTERT. The packaging cell line PT67 producing retroviruses containing hTERT was grown to 40% confluency and then cultured in KGM-2. After incubation at 37 °C for 48 h, the supernatant containing the retroviruses was filtered and stored at −80 °C. Isoprene diluted in KGM-2 was mixed with the retroviral solution and transferred to SV40 transfected KCs. After 24 h at 37 °C, the transduction solution was replaced by KGM-2 and incubated for 24 h. KCs were then cultured in KGM-2 supplemented with 50 μg/ml geneticindisulfat (G418, Sigma) at 37 °C and medium was changed every second to third day. Under this selection pressure only transduced KCs survived.

### Preparation of organotypic HSE

*In vitro* 3D HSE were generated as described previously^7^. Briefly, a suspension of collagen type I (Biochrom, Berlin, GER) containing 1 × 10^5^ fibroblasts per ml was poured into cell-culture inserts (3 μm pore size; BD Bioscience, Bedford, MA, USA) and allowed to gel for 2 h at 37 °C. After equilibration with KGM-2 for 2 h, 1.5 × 10^6^ KC, in a total volume of 2 ml KGM-2, were placed on the collagen gel. After overnight incubation the medium was removed from both the inserts and external wells, and serum-free KC-defined medium (SKDM) was added only to the external wells. SKDM consists of KGM-2 (without bovine pituitary extract and epinephrine) supplemented with 1.3 mM calcium chloride, 50 μg/ml ascorbic acid, and 0.1% bovine serum albumin (all supplements from Sigma-Aldrich). SKDM was changed every second day for 7 days.

For some experiments HSE were cultured in SKDM containing 10 µM of the DNA methylation inhibitor 5-Aza-2′-Deoxycytidine (Sigma Aldrich), 0.1 nM of proteasome inhibitor MG132 (Merck, Calbiochem, Darmstadt, Germany), 20 nM of the autophagy inhibitor bafilomycin A1 (InvivoGen, San Diego, CA, USA) or 80 mM of the p53 inhibitor pifithrin-α (Selleckchem, Munich, Germany).

### SDS–PAGE and Western blot

KC cultured in monolayer and epidermis samples from HSE were lysed in SDS–PAGE loading buffer, sonicated, centrifuged, and denatured with 0.1 M DL-Dithiothreitol (DTT, Sigma-Aldrich, Vienna, AUT) before loading. SDS–PAGE was conducted on 8–18% gradient gels (GE Amersham Pharmacia Biotech, Uppsala, SWE). The proteins were then electro-transferred onto nitrocellulose membranes (Bio-Rad, Hercules, CA, USA) and immunodetected with the primary and secondary antibodies listed in Tables 1 and 2. Reaction products were detected by chemiluminescence with the ImmunStar^TM^ Western C^TM^ Substrate kit (Bio-Rad) according to the manufacturer’s instructions. Ponceau S staining was done according to standard protocols and used as loading control. Full length blots of Fig. 6b are included in supplementary data in Figure S10.

### Protein microarray analysis

KC were lysed and analyzed using Proteome Profiler™ Human Phospho-Kinase Antibody Array (R&D Systems) according to the manufacturer’s instructions.

### Immunofluorescence labeling

Immunofluorescence labeling of 5 μm thin-sections of formalin-fixed, paraffin-embedded HSE, human abdominal and scalp skin was performed as described previously^7^. Briefly, after deparaffinization and rehydration, sections were heated in Target Retrieval Solution (Dako, Glostrup, Denmark) in the microwave at 450 W for 4 min. After washing with PBS (Gibco, Carlsbad, USA), sections were incubated with respective primary antibodies (Table 1) in PBS containing 2% BSA overnight at 4 °C. After washing, sections were incubated with the corresponding second step antibody (Table 2), 10% goat serum (Dako) and Hoechst (Life Technologies) in PBS containing 2% BSA for 30 min at room temperature and then the slides were mounted with Fluoprep (bioMérieux, Marcy l′Etoile, France). For Ki67 staining pre-blocking and primary antibody incubation was done in PBS containing 1% BSA, 5% goat serum and 0.2% Triton X. Target Retrieval Solution pH9 was used for antigen retrieval procedure of p53 and SV40 large T-antigen double immunofluorescence staining. Immunofluorescence labeling and transmission light images were recorded using the AX70 microscope with the imaging software MetaMorph from Olympus (Hamburg, GER).

### RNA isolation, cDNA synthesis and real-time PCR

RNA was isolated using RNeasy 96 Kit (Qiagen, Hilden, Germany) according to the manufacturer’s instructions. For cDNA synthesis RNA was reverse-transcribed with iScript cDNA Synthesis Kit (Bio-Rad, Hercules, CA, USA) and real-time PCR was carried out with LightCycler480 SYBR Green I Master (Roche Applied Science, Penzberg, Germany) according to the manufacturer’s instructions. The primers used are listed in Table 3, the relative expression of the target genes was calculated by comparing with the housekeeping gene Gapdh and experiments were performed in triplicate.

**Table 3:** List of used PCR primers.

Gene	Forward primer	Reverse primer
Gapdh	5′-CGAGATCCCTCCAAAATCAA-3′	5′-GGTGCTAAGCAGTTGGTGGT-3′
Keratin 5	5′-CAAGCGTACCACTGCTGAGA-3′	5′-TCAGCGATGATGCTATCCAG-3′
Keratin 10	5′-GCTGACCTGGAGATGCAAAT-3′	5′-AGCATCTTTGCGGTTTTGTT-3′
Filaggrin	5′-AAGGTTCACATTTATTGCCAAA-3′	5′-GGATTTGCCGAAATTCCTTT-3′
Loricrin	5′-GGAGTTGGAGGTGTTTTCCA-3′	5′-ACTGGGGTTGGGAGGTAGTT-3′
Tight junction protein 1	5′-TGCAAAGAGTGAACCACGAG-3′	5′-GGCACTTTTCCGAGATTCTG-3′
Claudin 1	5′-CCGTTGGCATGAAGTGTATG-3′	5′-CCAGTGAAGAGAGCCTGACC-3′
Occludin	5′-GATGAGCTGGAGGAGGACTG-3′	5′-GCAGATCCCTTCACTTGCTT-3′
Desmocollin 1	5′-GTGGTCAGCCTTTCGGTTTA-3′	5′-TTGGCAAATCCTGATCCTGT-3′
Transglutaminase 1	5′-GGTGAACTCCCTGGATGACA-3′	5′-AAGGGATGTGTCTGTGTCGT-3′
Small proline rich pr. 1 A	5′-GCCCATTCTGCTCCGTATAC-3′	5′-GGTTTTGGGGATGCATGGTT-3′
Small proline rich pr. 2 G	5′-CTCTCCACCACACTGATGCT-3′	5′-AATGCTCAGGTGGACAAGGA-3′
SPINK5	5′-ATGCACCAGGGAGCATAATC-3′	5′-TACAGGGGAGTCGTCCATTC-3′

Primers were synthesized by Microsynth (Balgach, Switzerland).

### Analysis of skin permeability

5 µl of LZ-linked biotin (ThermoScientific, Pierce, Waltham, MA, USA) were added onto the stratum corneum of the SE and incubated at 37 °C. After 60 min 6 mm punch biopsies were taken and samples were formalin-fixed and paraffin-embedded. 5 µm sections were prepared and biotin was labeled with Streptavidin conjugated to Alexa 594 (Invitrogen-Life Technologies, Carlsbad, CA, USA) and the nuclei were stained with Hoechst dye (Invitrogen-Life Technologies). The slides were mounted as described above.

### Chromosome preparation

Chromosome preparation was routinely performed as previously described^54^.

## Electronic supplementary material

Supplementary material

## Data Availability

Data, in anonymous format (according to data protection policy in the ethics agreement) is available on reasonable request.
