# Supplementary material for: Establishment of keratinocyte cell lines from human hair follicles
Source: Sci Rep. 2018 Sep 7;8:13434. doi: 10.1038/s41598-018-31829-0 (PMC6128885; doi:10.1038/s41598-018-31829-0)
Supplement: Supplementary file 1 — Supplementary material [file 41598_2018_31829_MOESM1_ESM.pdf]

Supplementary material

Establishment of keratinocyte cell lines from human hair follicles

Tanja Wagner[1$], Maria Gschwandtner[1$], Agata Strajeriu[2], Adelheid Elbe-Bürger[1], Johannes Grillari[2,3,4], Regina Grillari-Voglauer[2,3], Georg Greiner[5], Bahar Golabi[1], Erwin Tschachler[1*] and Michael Mildner[1*]

[1]Department of Dermatology, Medical University of Vienna, Vienna, Austria

[2]Evercyte, Vienna, Austria;

[3]Department of Biotechnology, University of Natural Resources and Life Sciences, Vienna, Austria

[4]Christian Doppler Laboratory for Biotechnology of Skin Aging, Vienna, Austria

[5]Department of Laboratory Medicine, Medical University of Vienna, Vienna, Austria

$ These authors share first authorship

* These authors share last and corresponding authorship

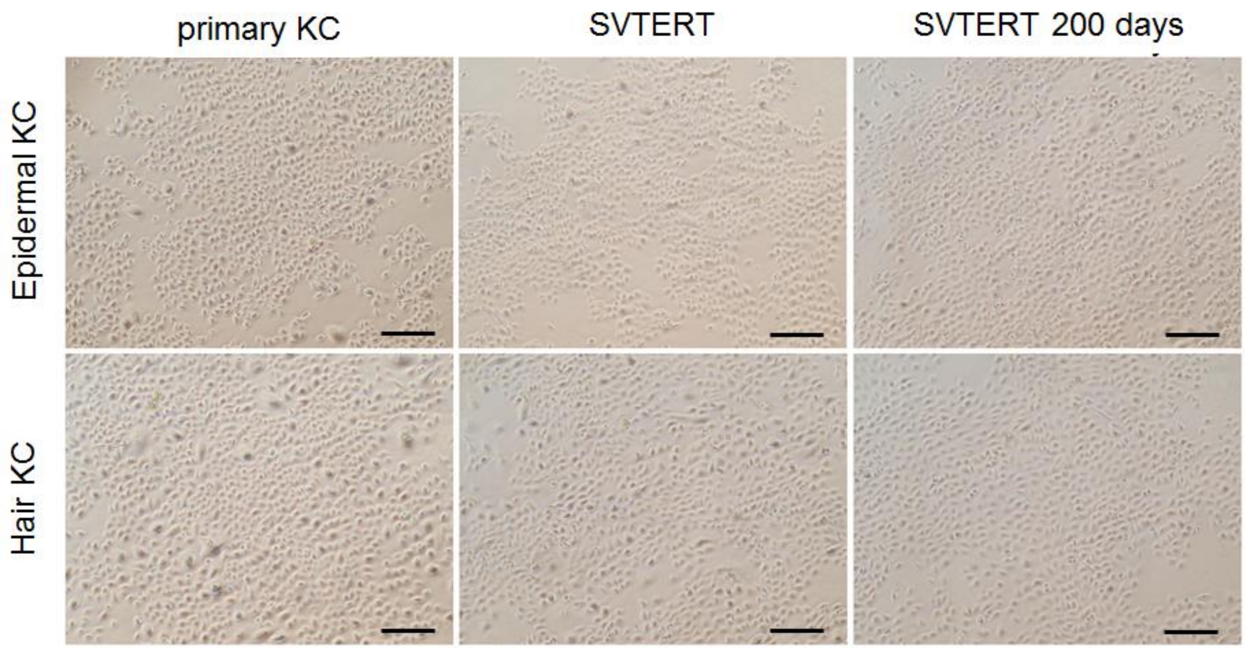

Supplementary Figure 1: The morphology of SVTERT KC does not differ from primary KC. Primary and immortalized KC derived from epidermis and hair show similar morphology. Phase contrast pictures are shown. Scale bar = 240 µm.

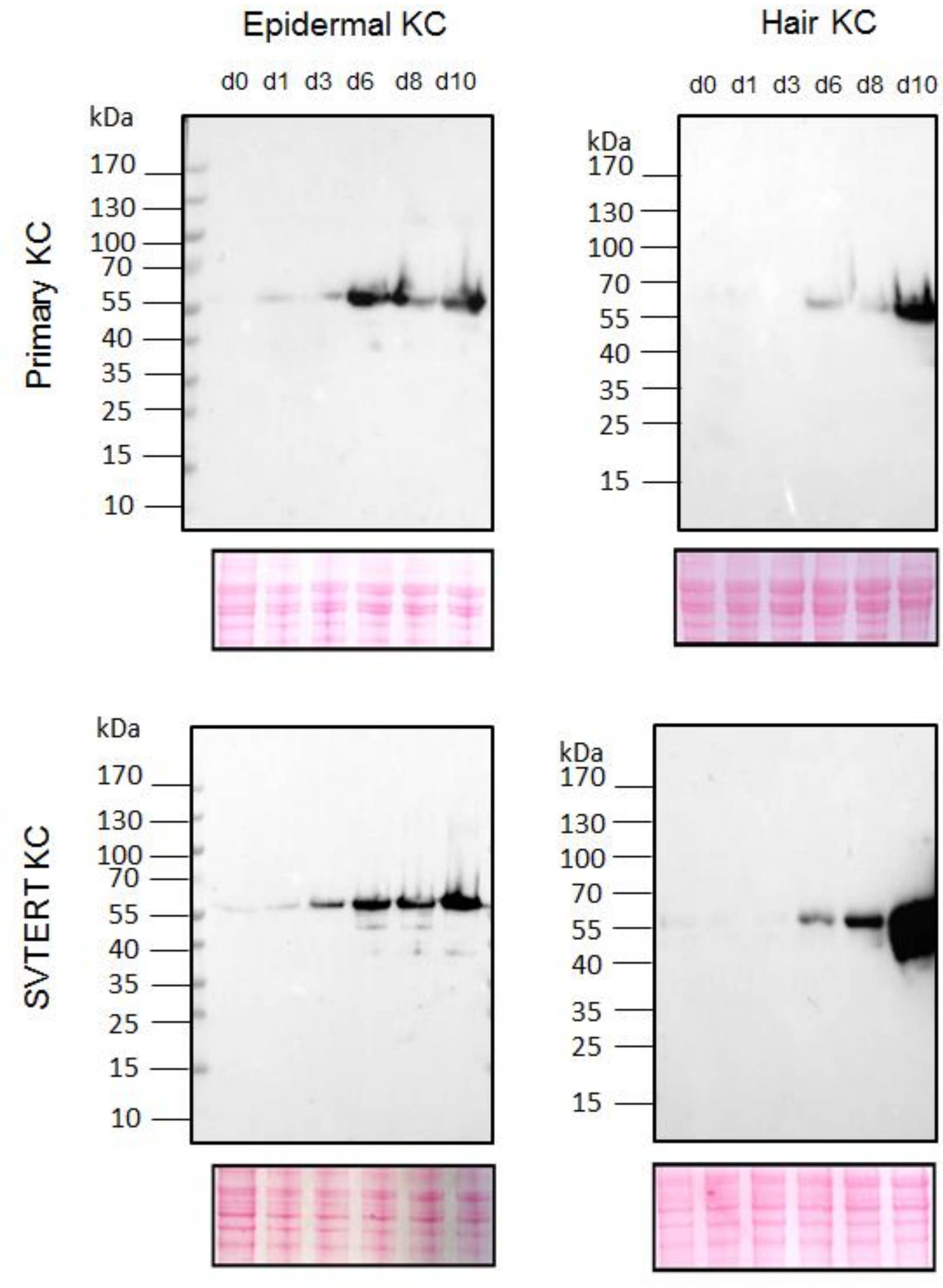

Supplementary figure 2: Primary and SVTERT KC differentiate in monolayer culture.
Primary and SVTERT KC derived from epidermis and hair were cultured postconfluent for ten days and the expression of the differentiation-associated protein (KRT10) was analyzed by Western Blot at indicated time points. Primary and SVTERT epidermal KC showed significant induction of KRT10 starting at day 3. Primary and SVTERT hair KC showed significant induction of KRT10 starting at day 6. Representative Western Blots and Ponceau S stainings (as loading control) of three independent experiments are shown.

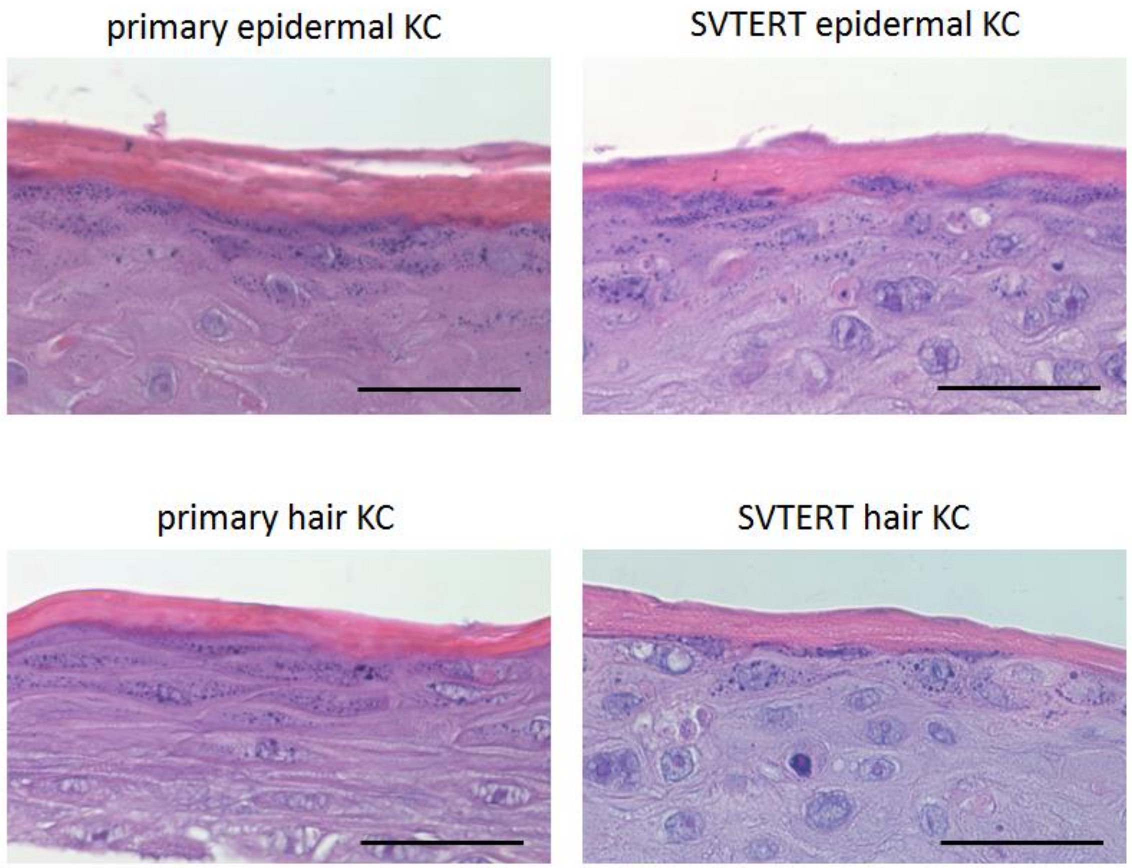

Supplementary Figure 3: Skin equivalents with primary and SVTERT KC show similar structure of the stratum granulosum.
High magnification images of hematoxylin and eosin stained fully developed human skin equivalents (HSE) show no difference in the phenotype in the granular layer of HSE done with primary or SVTERT KC. Scale bar = 60 µm.

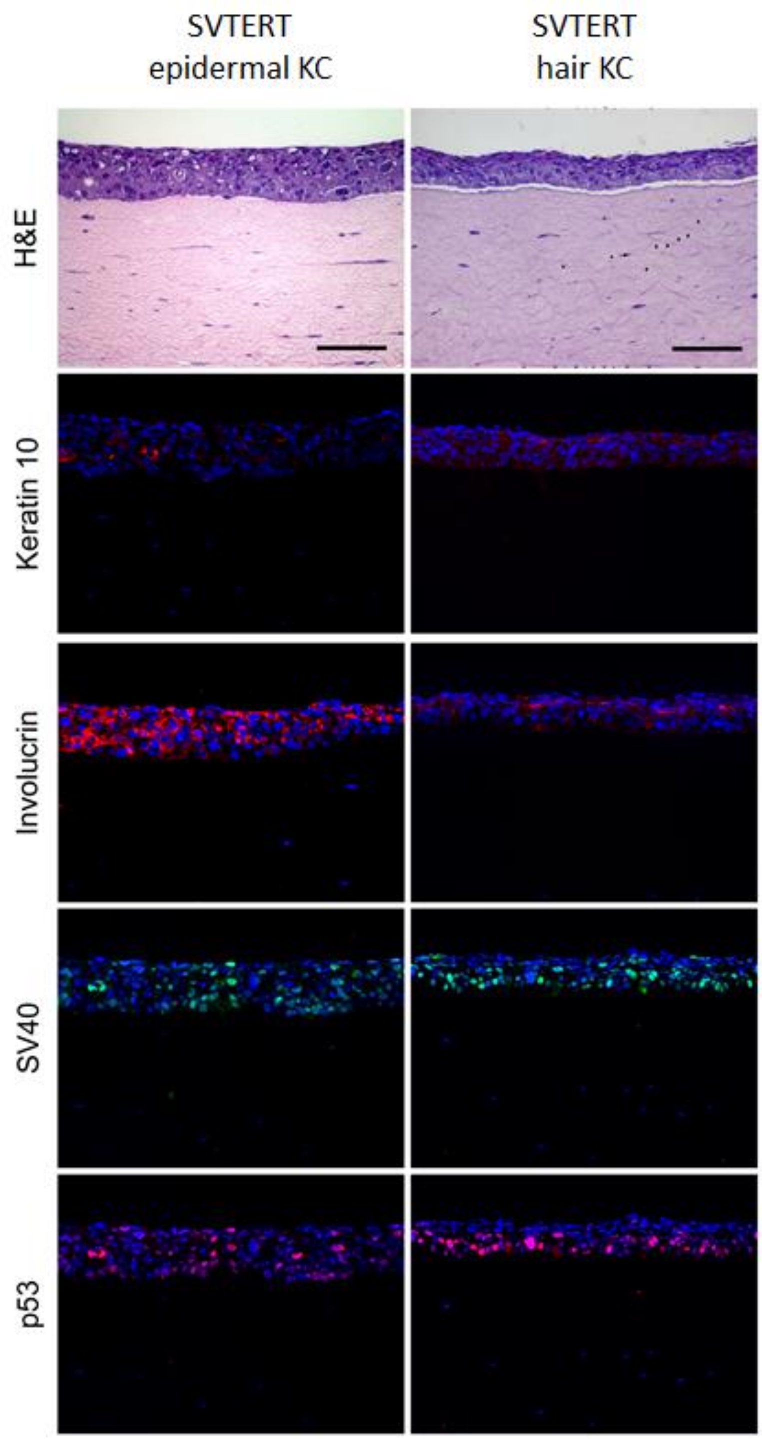

Supplementary Figure 4: SVTERT KC cultured for ≥ 200 days do not form a fully differentiated skin equivalent.
Human skin equivalents (HSE) with SVTERT epidermal and hair-derived KC were analyzed at day seven by hematoxylin and eosin (H&E) and immunfluorescence staining. HSE developed a multilayered epidermis, but no intact stratum granulosum and stratum corneum. The differentiation associated protein KRT 10 was hardly detectable and involucrin showed an abnormal expression pattern. SV40 large T-antigen and p53 expression was detected in all epidermal layers. One representative experiment out of three is shown; Scale bar = 120 µm.

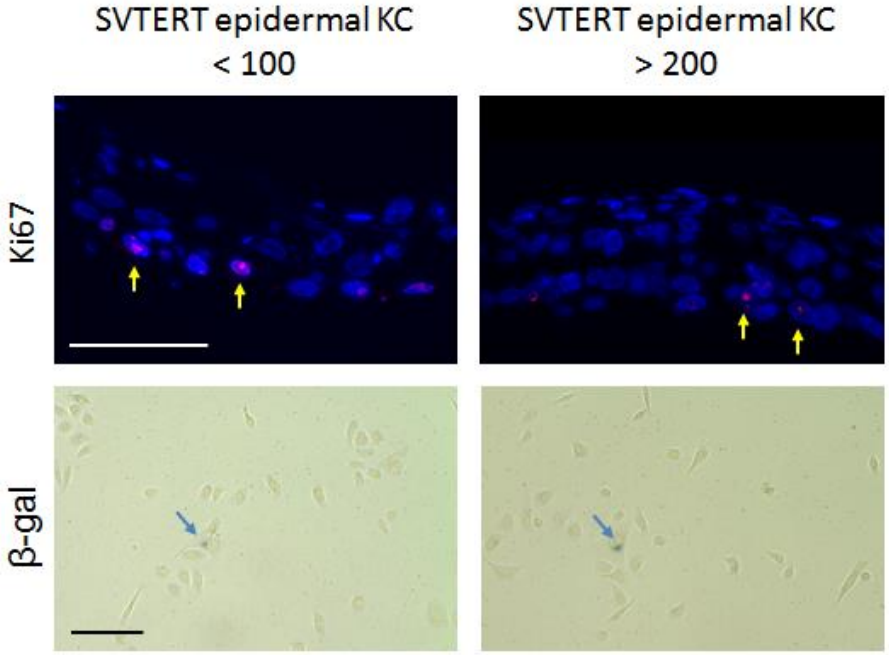

Supplementary Figure 5: No changes in Ki67 and β-galactosidase staining in SVTERT KC cultured for ≥ 200 days.
Human skin equivalents (HSE) with primary and SVTERT epidermal KC were were analyzed for Ki67 at day seven with immunofluorescence staining and β-galactosidase was stained in monolayer cultured KC. No differences in SVTERT epidermal KC cultured for <100 doublings and >200 doublings were observed. Yellow and blue arrows indicate Ki67 and β-galactosidase positive cells, respectively. Scale bar = 120 µm.

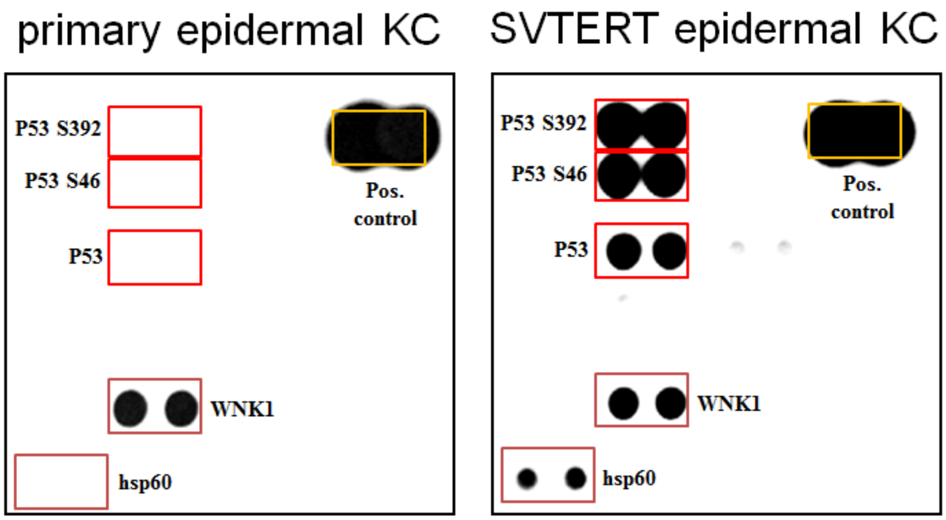

Supplementary Figure 6: Upregulation of p53 in SVTERT epidermal KC
Human skin equivalents (HSE) with primary and SVTERT epidermal KC were were analyzed at day seven with a Phospho-Kinase Antibody Array (R&D Systems). p53 was not detected in primary KC, but was highly upregulated in SVTERT KC.

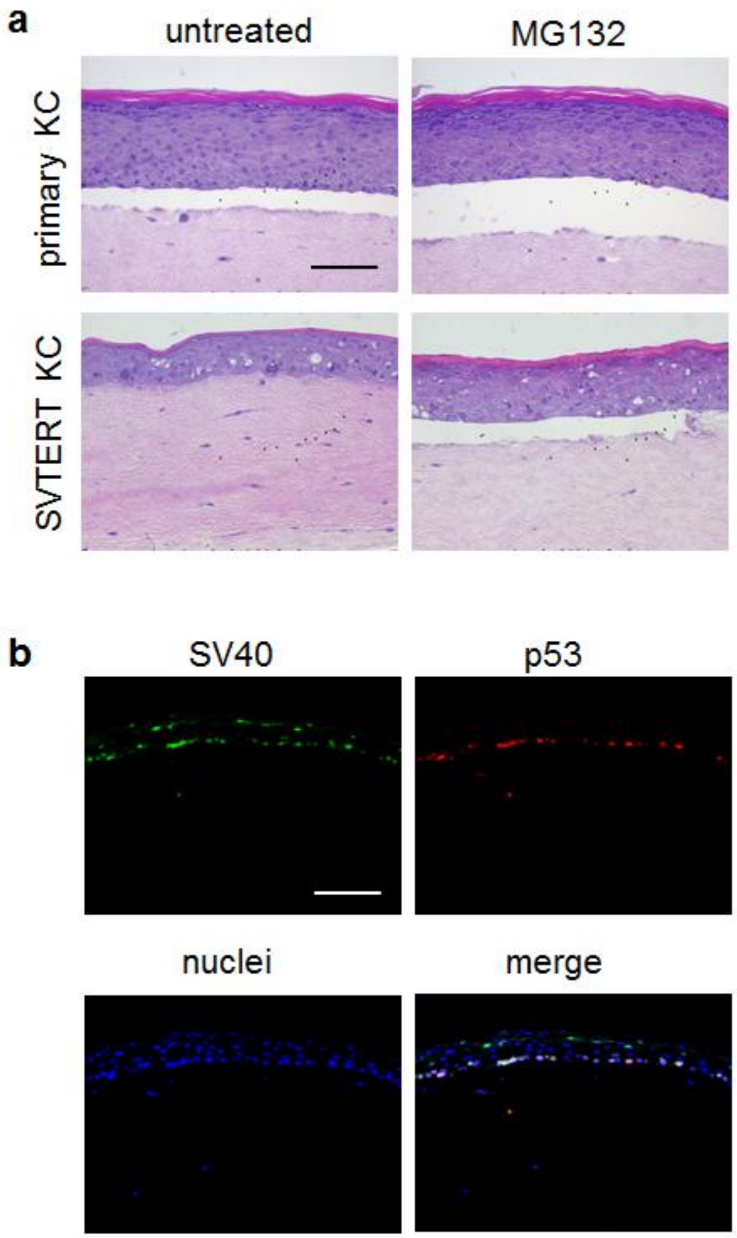

Supplementary Figure 7: The proteasome inhibitor MG132 does not influence SV40 large T-antigen and p53 expression in skin equivalents.
Human skin equivalents (HSE) with primary and SVTERT epidermal KC were analyzed at day seven by hematoxylin and eosin (H&E) and immunfluorescence staining. 0.1 nM of the proteasome inhibitor MG132 did not modulate architecture of the HSE (a) and the expression of SV40 large T-antigen and p53 in HSE with SVTERT KC was similar to untreated controls (b; compare to Figure 6 for primary KC). Scale bar = 120 µm.

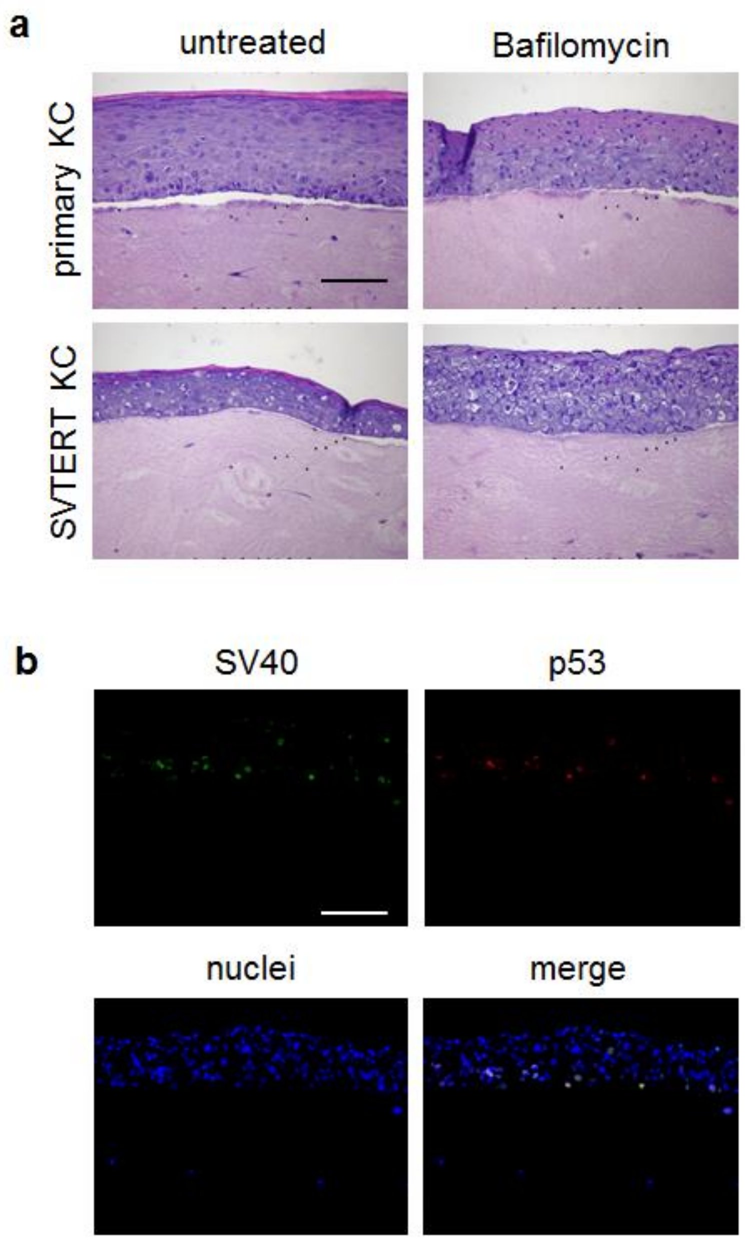

Supplementary Figure 8: The lysosome/autophagy inhibitor bafilomycin A1 does not modulate SV40 large T-antigen and p53 expression in skin equivalents.
Human skin equivalents (HSE) with primary and SVTERT epidermal KC were analyzed at day seven by hematoxylin and eosin (H&E) and immunfluorescence staining. 20 nM of the autophagy inhibitor bafilomycin A1 resulted in irregular architecture of the HSE including parakeratosis (a), but the expression of SV40 large T-antigen and p53 in HSE with SVTERT KC was similar to untreated controls (b; compare to Figure 6 for primary KC). Scale bar = 120 µm.

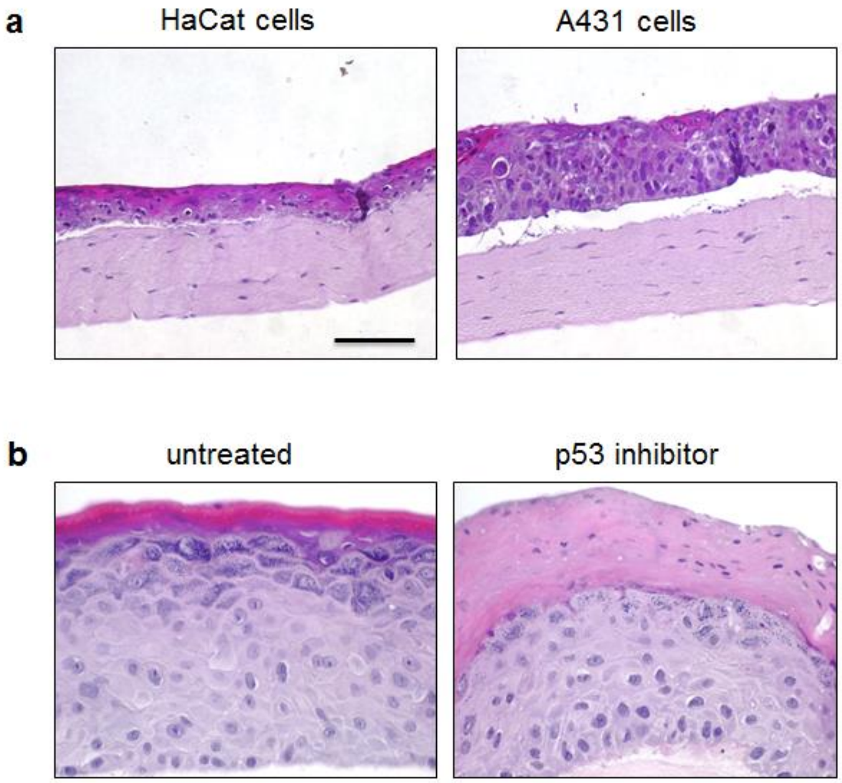

Supplementary Figure 9: p53 expression is important for the formation of fully differentiated skin equivalents.
Human skin equivalents (HSE) with HaCaT and A431 cells (a) and primary epidermal KC (b) were analyzed at day seven by hematoxylin and eosin (H&E) staining. Neither HaCaT nor A431 cells gave rise to a fully differentiated HSE (a). 80 mM of the p53 inhibitor Pifithrin-α resulted in incomplete differentiation and parakeratosis of SE from primary epidermal KC (b). Scale bar = 120 µm.

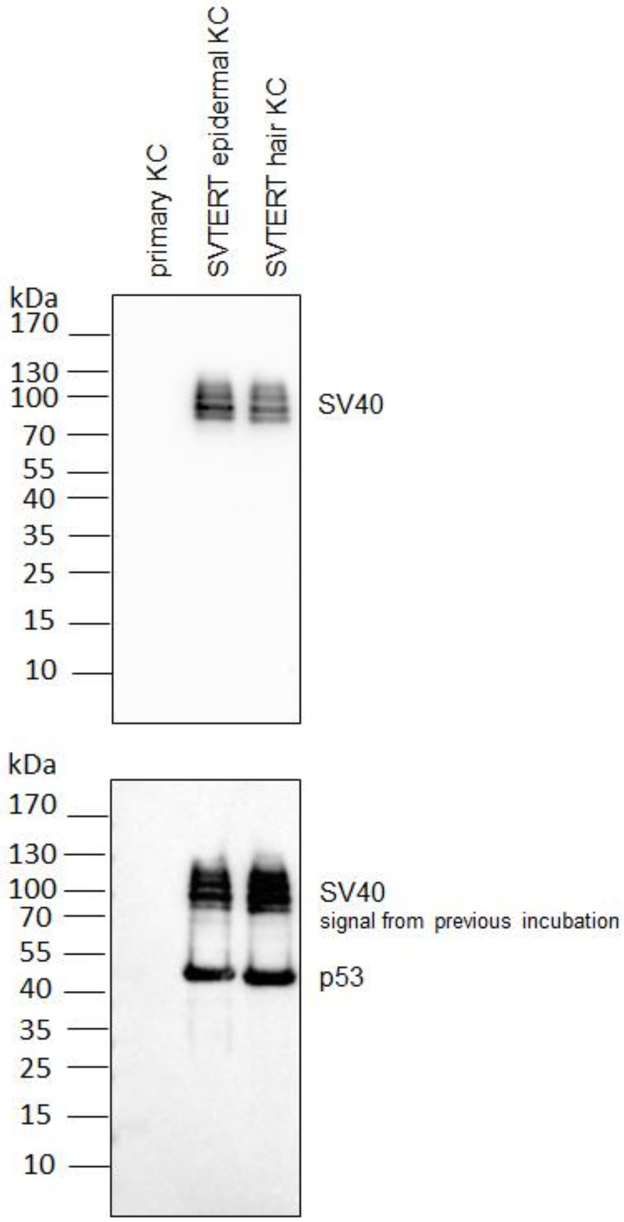

Supplementary Figure 10: Full length blots of data presented in Figure 6b (Skin equivalent cultures with SVTERT KC show p53 expression limited to the basal layer).
HSE cultures from primary and SVTERT skin and hair KC were established and analyzed at day seven by Western Blot. p53 and SV40 large T-antigen expression was detected in SVTERT epidermal and hair KC, but not in primary KC. p53 antibody was re-incubated on a membrane that was detected with SV40 beforehand. Representative Western Blots of three independent experiments are shown.
